# Accuracy of the Short-Form Montreal Cognitive Assessment Chinese Versions

**DOI:** 10.3389/fnagi.2021.687824

**Published:** 2021-06-22

**Authors:** Ji-ping Tan, Xiaoxiao Wang, Shimin Zhang, Yiming Zhao, Xiaoyang Lan, Nan Li, Lu-ning Wang, Jing Gao

**Affiliations:** ^1^Geriatric Neurology Department of The Second Medical Center & National Clinical Research Center for Geriatric Diseases, Chinese PLA General Hospital, Beijing, China; ^2^Research Center of Clinical Epidemiology, Peking University Third Hospital, Beijing, China; ^3^Neurology Department of The First Medical Center, Chinese PLA General Hospital, Beijing, China; ^4^Department of Neurology, Peking Union Medical College Hospital, Beijing, China

**Keywords:** MOCA, s-MoCA, cognitive impairment, item response theory, dementia

## Abstract

**Background:** There is a strong need for short and effective methods to screen for cognitive impairment. Recent studies have created short forms of the Montreal Cognitive Assessment (s-MoCA) in English-speaking populations. It is also important to develop a validated Chinese short version to detect cognitive impairment.

**Methods:** Item response theory and computerized adaptive testing analytics were used to construct abbreviated MoCAs across a large neurological sample comprising 6,981 community-dwelling Chinese veterans.

**Results:** Six MoCA items with high discrimination and appropriate difficulty were included in the s-MoCA. The Chinese short versions (sensitivity 0.89/0.90, specificity 0.72/0.77) are similar in performance to the full MoCA in identifying cognitive impairment (sensitivity 0.91, specificity 0.82).

**Conclusions:** These short variants of the MoCA may serve as quick and effective instruments when the original MoCA cannot be feasibly administered in clinical services with a high patient burden and limited cognitive testing resources.

## Background

Dementia is the most common disabling neurological disease in people older than 65 years and affects ~10 million people in China (GBD 2016 Dementia Collaborators, 2019). This number accounts for ~25% of the entire population with dementia worldwide (GBD 2016 Dementia Collaborators, 2019). Dementia places a heavy burden on patients, caregivers, communities, and societies (Jia et al., 2020). While there is no cure for dementia, the early identification of cognitive impairment enables the identification and management of modifiable risk factors and can slow the progression of the disease (Eshkoor et al., 2015). Therefore, there is a strong need for adequate methods to screen for cognitive impairment.

Many instruments used for cognitive impairment screening are available. The Mini-Mental State Examination (MMSE) (Folstein et al., 1975) and Montreal Cognitive Assessment (MoCA) (Nasreddine et al., 2005) are two of the most common instruments. These tests can be easily administered to participants and demonstrate diagnostic utility (Wang and Dong, 2018). Compared with the MMSE, the MoCA is significantly better in the detection of mild cognitive impairment (MCI) among people aged over 60 (Tan et al., 2015; Pinto et al., 2019), and it also displays more effectiveness in detecting dementia (Tan et al., 2015). In addition, the MoCA is more sensitive in identifying MCI and mild dementia (Tan et al., 2015). Thus, the MoCA is widely used worldwide.

The MoCA takes ~10–15 min to administer (Roalf et al., 2016). However, this is too long for initial screening in busy routine healthcare visits. Moreover, an abbreviated MoCA that maintains its diagnostic accuracy may have particular utility because the elderly population (particularly the oldest group) may struggle to complete the full MoCA. In addition, there seems to be a strong need for a short cognitive test that can be conducted with visually or hearing-impaired populations, as well as other clinical populations (stroke patients). This is critical because relatively short-form versions of the MoCA (s-MoCA) would ease administration of the test in these populations compared with the full MoCA.

Several different abbreviated versions of the MoCA have been identified by systematic literature searches (Liew, 2019; McDicken et al., 2019). These brief versions with differing content and test properties suffer from small sample sizes, limited applicability to clinical samples [vascular dementia (VD) or Alzheimer's disease (AD) only], less rigorous methodology used to derive the s-MoCA and suboptimal methods for the clinical diagnosis of dementia. Consequently, it is challenging to accurately generalize these short forms to other neurological samples (particularly elderly community participants). Moreover, these abbreviated MoCAs were created for English-speaking populations. Therefore, it is necessary to develop a validated Chinese short version to detect cognitive impairment in the elderly Chinese population.

Here, we aimed to develop a short-form MoCA Chinese version (s-MoCA-CHN) to detect MCI and dementia. In this study, elderly Chinese veterans were recruited and underwent cognitive screening, neuropsychological testing and clinical diagnosis. Item response theory (IRT) (Nguyen et al., 2014) and IRT-based computerized adaptive testing (CAT) (Nguyen et al., 2014) analytics were used to construct abbreviated versions of the MoCA. In addition, the performance of these brief forms across education levels and age groups was examined.

## Methods

### Study Population

The Institutional Review Board of the General Hospital of the People's Liberation Army approved this study, and written informed consent was obtained. We reviewed our database of participants, who were prospectively identified and recruited with the help of the Chinese Veteran Clinical Research (CVCR) Platform for the assessment of non-communicable diseases. Details regarding the CVCR design have been reported elsewhere (Tan et al., 2014). The Chinese veteran population is predominantly male. Of the 11,593 people who met the inclusion criteria, 9,676 elderly Chinese veterans were recruited into this platform. Among the 9,676 veterans who were approached, 7,445 veterans had complete neuropsychological test and clinical diagnosis data. The MoCA data of 356 participants (4.8%) were missing. The demographic information, including age, gender, and education, of 148 participants (2.0%) was missing. Data from 6,981 veterans were included in the analysis after these missing data were excluded.

Extensively uniformly trained and qualified medical staff from the Departments of Neurology and Geriatrics performed a two-stage investigation screening and diagnostic assessment. All participants were Chinese speaking and were screened using the Chinese version of the MMSE (Zhang et al., 1999) and a revised Chinese version of the MoCA (Tan et al., 2015) (the Peking Union Medical College Hospital version of the MoCA). The participants completed a neuropsychological battery designed for the assessment of memory, language, visuospatial perception, calculation, abstract reasoning, and executive function. The clinical diagnosis was made by consensus based on the patient's history, neuropsychological tests, physical and neurological examinations, neuroimaging and laboratory data. MCI was diagnosed according to the core clinical criteria recommended by the National Institute on Aging and the Alzheimer's Association Working Group (Albert et al., 2011). Dementia was diagnosed according to the Diagnostic and Statistical Manual of Mental Disorders IV (American Psychiatric Association, 1994). The index test participants were blinded to the clinical information and standard reference results.

### Item Calibration and Computerized Adaptive Test Simulation

IRT and CAT analytics were performed to develop the short form of the Chinese version of the MoCA. IRT enables the design or improvement of instruments by determining the items' discrimination and difficulty. The graded response model (GRM), which is capable of addressing dichotomous and polytomous items on the same test, was used to obtain the item parameter estimates and ability estimates, which were then used in CAT. In the MoCA, the items trail making, cube copy, attention (number) and language fluency are scored dichotomously (correct/incorrect), and the other items are scored polytomously.

An exploratory factor analysis was used to test for sufficient unidimensionality for IRT. The following two criteria were used (Nguyen et al., 2014): (1) the first factor accounted for at least 20% of the variability and (2) the ratio of the first to the second factor was >4. To test for the local independence assumption of IRT, we examined the residual correlation matrix produced by confirmatory factor analysis (CFA). High residual correlations (>0.2) were flagged, and the local independence assumption was violated (Nguyen et al., 2014). The monotonicity assumption was evaluated graphically by plotting characteristic curves for each item. The assumption indicates that as a person's ability increases, the probability of providing the correct answer also increases (Nguyen et al., 2014).

CAT (Nguyen et al., 2014) was applied to the standard MoCA to determine which items to include in the s-MoCA. Initially, an item was administered based on the examinee's ability estimate. CAT successively selects items based on the examinee's performance on previous items. For example, if an examinee responds correctly to an item of average difficulty, the examinee's theta estimate is increased, and a more difficult question is then presented. If an examinee performs poorly, the examinee's theta estimate is decreased, and the examinee is presented with a simpler question. This process continues until the stopping criterion is reached. In this study, the CAT simulations stopped when the examinee's standard error of measurement (SEM) was <0.3 or a maximum of eight items were administered. The usefulness of the items was judged based on how often they were administered in the CAT simulations.

### Statistical Analyses

The IRT models were estimated using the ltm package in R version 4.0.2. IRT-based CAT was simulated using firestar 1.5.1 (Choi, 2009). The statistical analyses were performed using IBM SPSS Statistics 26.0 and NCSS 12.0. Continuous variables are expressed as medians and interquartile ranges (IQRs), and categorical variables are expressed as percentages. The Mann-Whitney *U*-test and Kruskal-Wallis *H*-test were used to examine the differences between the groups. The Spearman correlation coefficient was used to evaluate the relationship between the s-MoCA and the standard MoCA. We examined the difference between the areas under the receiver operating characteristic curve (AUC) using the DeLong test. The AUC values range from 0 to 1, with scores closer to 1 representing higher predictive accuracy. The level of significance was set at α = 0.05.

### Data Availability Statement

The authors confirm that the data supporting the findings of this study are available within the article and its Supplementary Materials.

## Results

### IRT Assumptions

The ratio of the first to second eigenvalues (4.05) and the magnitude of the first eigenvalue (36.7% of the variability of the first factor) indicated that the MoCA is sufficiently unidimensional. The examination of the residual correlation matrix (residual correlations <0.2) resulting from the single-factor CFA was supportive of the local independence assumption (see online Supplementary Figure 2). The item characteristic curves indicated that the probability of endorsing an item increased as an individual's trait level increased (see online Supplementary Figure 3), indicating the assumption of monotonicity.

### Derivation of the Chinese s-MoCA

The IRT and CAT of the MoCA across a large sample identified six items with high discrimination and sufficiently variable difficulties in discriminating between healthy individuals and individuals with cognitive impairment (MCI and dementia). The selected items included clock draw, digit span, serial subtraction, delayed recall, abstraction, and trail making (Table 1).

**Table 1:** Item usage and selection in the CAT for s-MoCA (*n* = 6,981).

**MoCA item**	**Domain**	**Score range**	**Usage (%)**	**s-MoCA**	
				**4-item (0–13)**	**6-item (0–16)**
Trail making	Visual/executive	0–1	90		Y
Cube copy	Visual/executive	0–1	71		
Clock draw	Visual/executive	0–3	100	Y	Y
Naming (lion, cow, camel)	Naming	0–3	6		
Digit span (backwards, forwards)	Attention	0–2	100	Y	Y
Attention (number)	Attention	0–1	17		
Serial subtraction	Attention	0–3	100	Y	Y
Language repetition	Language	0–2	79		
Language fluency	Language	0–1	11		
Abstraction (train, watch)	Abstraction	0–2	93		Y
Recall	Recall	0–5	100	Y	Y
Orientation (day, month, year, week, place, city)	Orientation	0–6	34		

*CAT, Computerized Adaptive Test; MoCA, Montreal Cognitive Assessment; s-MoCA, short forms of the standard MoCA; Y, Yes*.

Using these items, two versions of the s-MoCA consisting of 4 or 6 items were constructed. The 4-item s-MoCA (score range: 0–13) consisted of the following four core items: clock draw, digit span, serial subtraction, and recall. Both abstraction and trial making were added to construct a 6-item version of the s-MoCA (score range: 0–16). There was a strong positive correlation between these brief versions of the s-MoCA and the standard MoCA (Spearman *r* = 0.915 and 0.963, *p* < 0.001).

### Performance of the s-MoCA in Broad Neurological Samples

Participants were 6676 male (95.6%) and 305 female (4.4%) veterans aged 60 years and older. Among 6,981 elderly veterans, 102 (1.5%), 2,056 (29.5%), 4,646 (66.6%), and 177 (2.5%) veterans were in the four age groups (60–69, 70–79, 80–89, and ≥90 years old), respectively. 2,649 (37.9%), 3,179 (45.5%), and 1,153 (16.5%) elderly veterans were categorized into three education groups (<7, 7–12, and >12 years of education). After a thorough assessment, participants were classified into the following diagnostic groups: the cognitively healthy controls (HCs, *n* = 4,007), MCI group (*n* = 2,205), and dementia group (*n* = 769).

In this study, the s-MoCA and standard MoCA scores followed the same pattern (Table 2). The s-MoCA scores of the affected individuals were significantly lower than those of the HCs. The amnestic MCI (aMCI) participants and non-amnestic MCI (naMCI) participants both had lower scores than the HCs (*p* < 0.001). In addition, the dementia group (AD, VD, and other types) had lower s-MoCA scores than the HC group (*p* < 0.001). The s-MoCA scores increased with higher education and decreased with advancing age (*p* < 0.001). The differences in the s-MoCA scores among the different dementia subtypes (AD, VD, and others) were weak and non-significant (*p* = 0.111 and 0.132, respectively). In the MCI sample, the presence or absence of memory impairment had no effect on the s-MoCA scores (*p* = 0.521 and 0.212, respectively).

**Table 2:** MoCA and s-MoCA in a broad neurological sample (*n* = 6,981).

	**MoCA** ** (0–30)**	***p***	**s-MoCA 4-item** ** (0–13)**	***p***	**s-MoCA 6-item** ** (0–16)**	***p***
Neurological diagnoses		<0.001		<0.001		<0.001
Normal (*n* = 4,007)	27 (26–28)		11 (10–12)		14 (13–15)	
MCI (*n* = 2,205)^a^	22 (20–24)		8 (7–9)		9 (8–11)	
Dementia (*n* = 769)^a^	16 (11–20)		5 (3–7)		6 (3–8)	
MCI		0.180		0.521		0.212
Amnestic (*n* = 1,204)^a^	22 (19–24)		8 (7–9)		9 (8–11)	
Non-amnestic (*n* = 1,000)^a^	22 (20–24)		8 (7–10)		10 (8–11)	
Dementia		0.023		0.111		0.132
AD (*n* = 476)^a^	16 (12–20)		5 (3–7)		6 (4–9)	
VD (*n* = 151)^a^	15 (9–20)		5 (2–8)		6 (2–9)	
Other (*n* = 142)^a,b^	15 (8–19)		5 (2–7)		6 (2–8)	
Age^c^		<0.001		<0.001		<0.001
<80 yrs. (*n* = 2,158)	27 (25–28)		11 (9–12)		13 (12–15)	
80–89 yrs. (*n* = 4,646)	25 (21–27)		10 (7–11)		12 (9–14)	
≥90 yrs. (*n* = 177)	21 (15–26)		7 (5–11)		9 (6–13)	
Education^c^		<0.001		<0.001		<0.001
<7 yrs. (*n* = 2,649)	25 (20–27)		10 (7–11)		12 (8–14)	
7–12 yrs. (*n* = 3,179)	26 (22–28)		10 (8–12)		13 (10–14)	
>12 yrs. (*n* = 1,153)	27 (24–28)		11 (9–12)		13 (11–15)	

a *MoCA and s-MoCA scores were significantly lower than those of HCs*.

b *MoCA scores were significantly lower than those of AD*.

c *Pairwise comparisons were statistically significant*.

*MoCA, Montreal Cognitive Assessment; s-MoCA, short forms of the standard MoCA; MCI, mild cognitive impairment; AD, Alzheimer's disease; VD, vascular dementia*.

### Potential of the s-MoCA to Detect Cognitive Impairment

**Figure 1:**
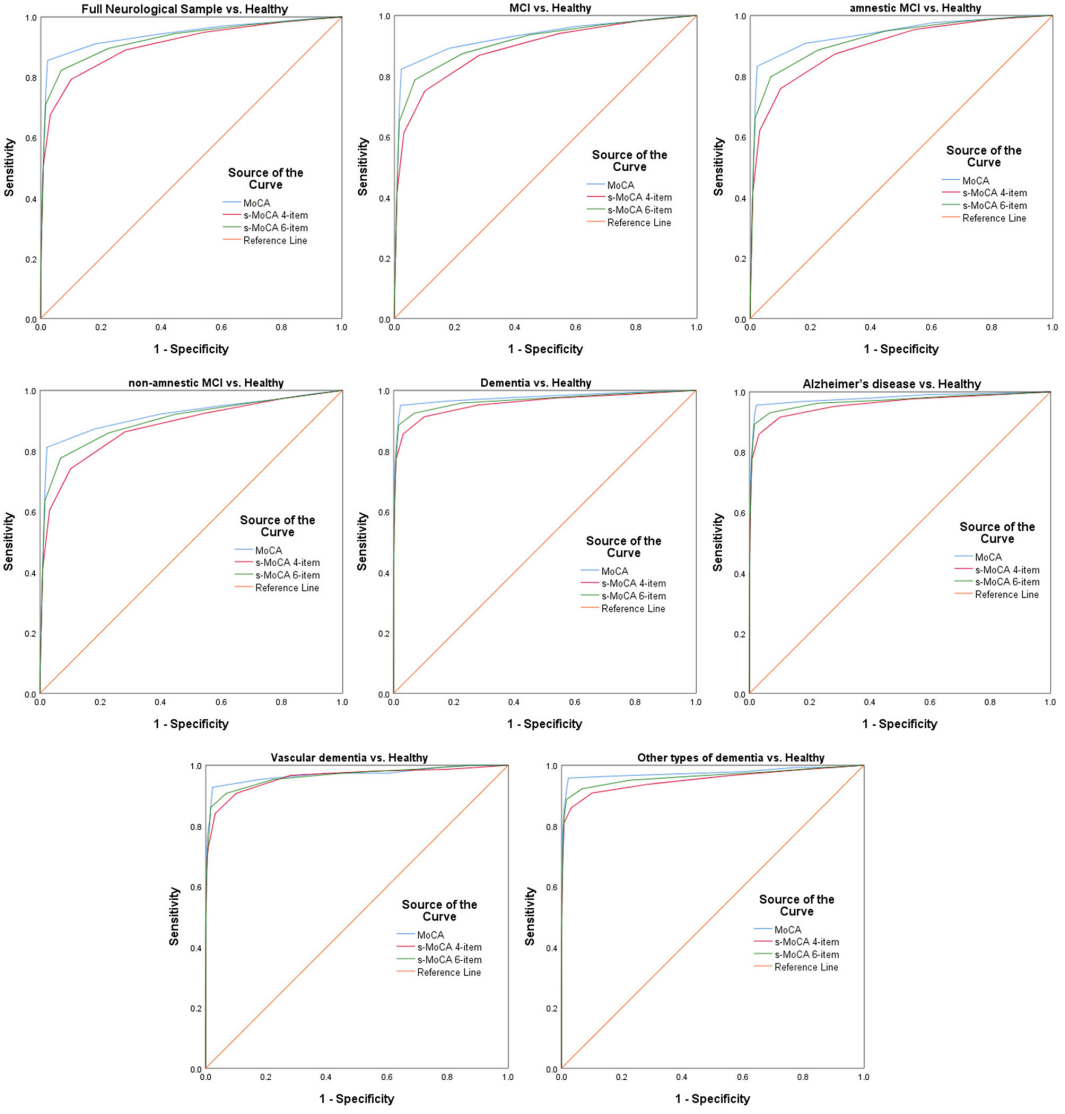
Receiver operating characteristic curves of the standard MoCA, 4-item s-MoCA and 6-item s-MoCA; MoCA, Montreal Cognitive Assessment; s-MoCA, short-form versions of the MoCA.

**Figure 2:**
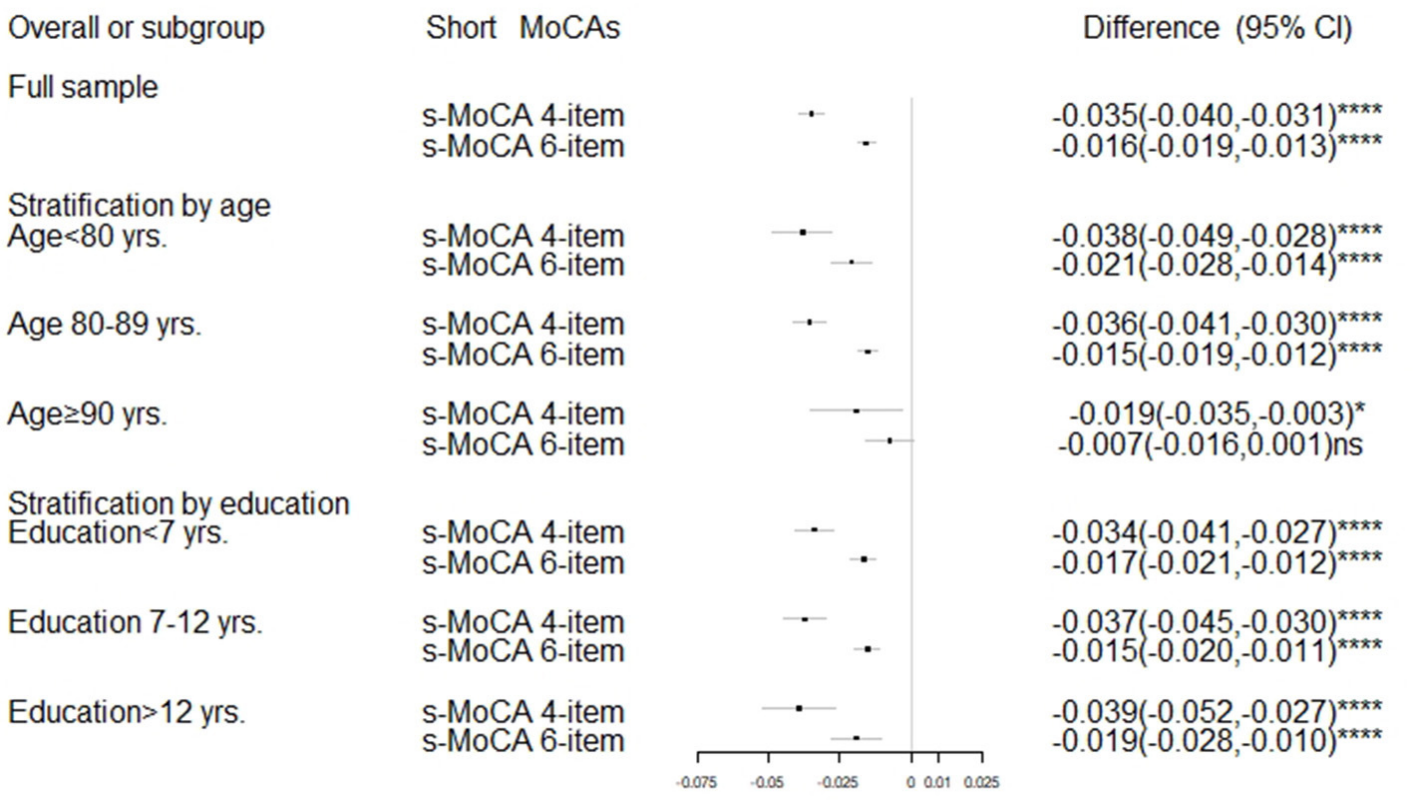
The difference of the AUC for s-MoCA minus the AUC for MoCA. MoCA, Montreal Cognitive Assessment; s-MoCA, short form versions of the MoCA; AUC, areas under receiver operating characteristic curves; CI, confidence interval; ****p < 0.0001; *p < 0.05; ns: *p* > 0.05; *P*-value was based on the DeLong test, a method to compare two AUCs.

The MoCA and s-MoCA had an AUC ≥ 0.88 when comparing different types of MCIs with HCs and an AUC ≥ 0.95 when comparing different types of dementia with HCs, demonstrating that the full MoCA and s-MoCA tests had high diagnostic accuracy among individuals with varying cognitive function (Figure 1). In the full sample, the AUC of the standard MoCA comparing affected individuals (MCI and dementia) with healthy individuals was 0.942. The AUCs of these two versions of the s-MoCA were high (0.906 and 0.926) but slightly lower than the AUC of the standard MoCA (*z* = 14.894, *p* < 0.001 in the comparison of MoCA and the 4-item s-MoCA; *z* = 10.561, *p* < 0.001 in the comparison of MoCA and the 6-item s-MoCA). This pattern was observed in most subgroups, and the difference of the AUC for s-MoCA minus the AUC for MoCA varied from −0.039 to −0.015 (Figure 2). In addition, the 6-item s-MoCA demonstrated comparable performance (0.941) to the original MoCA (0.949) in the oldest-old group (*z* = −1.758, *p* = 0.079).

In addition, old-old individuals exhibited outperforming scores on these two forms of the s-MoCA, with higher AUC values than those of individuals younger than 80 years (0.908 vs. 0.886, *z* = 2.233, *p* = 0.026 on the 4-item s-MoCA; 0.928 vs. 0.903, *z* = 1.841, *p* = 0.007 on the 6-item s-MoCA). The oldest-old group had higher AUC values in the two short forms of the MoCA (0.929 and 0.941, respectively), but these differences were not significantly different from those of the other two age groups (*p* > 0.05).

The less-educated participants performed better on these two forms of the s-MoCA, with significantly higher AUC values (0.921 and 0.939, respectively) than those of the individuals with ≥7 and ≤ 12 years of education (0.897 and 0.919, respectively) and individuals with >12 years of education (0.883 and 0.903, respectively).

The optimal cutoff score of the standard MoCA was ≤ 25, with no adjustments for education in differentiating individuals with MCI and dementia from HCs. The diagnostic accuracy was high (sensitivity 0.91, specificity 0.82). Correspondingly, the optimal cutoff scores of these short MoCA tests were ≤ 10 and ≤ 12. These tests demonstrated a sensitivity of 0.89/0.90 and specificity of 0.72/0.77. There was satisfactory diagnostic accuracy in the different age and education groups (Table 3).

**Table 3:** Diagnostic accuracy of the MoCA and s-MoCA for cognitive impairment (MCI +dementia).

	**MoCA (0-30)**, **≤25**				**s-MoCA 4-item (0-13)**, **≤10**				**s-MoCA 6-item (0-16)**, **≤12**			
	**Se/Sp**	**AUC (95%CI)**	***p***	***P***	**Se/Sp**	**AUC (95%CI)**	***p***	***p***	**Se/Sp**	**AUC (95%CI)**	***p***	***p***
Full sample (*n* = 6,981)	0.91/0.82	0.942 (0.935–0.948)	—	—	0.89/0.72	0.906 (0.899–0.914)	—	—	0.90/0.77	0.926 (0.919–0.933)	—	—
**Age**												
<80 yrs. (*n* = 2,158)	0.87/0.87	0.924 (0.908–0.939)	Ref	—	0.85/0.76	0.886 (0.868–0.903)	Ref	—	0.84/0.82	0.903 (0.886–0.920)	Ref	—
80–89 yrs. (*n* = 4,646)	0.92/0.79	0.943 (0.936–0.950)	0.025	Ref	0.90/0.70	0.908 (0.899–0.916)	0.026	Ref	0.91/0.75	0.928 (0.920–0.936)	0.007	Ref
≥90 yrs. (*n* = 177)	0.96/0.80	0.949 (0.909–0.988)	0.251	0.792	0.92/0.72	0.929 (0.886–0.973)	0.066	0.331	0.94/0.74	0.941 (0.900–0.983)	0.094	0.546
**Education**												
<7 yrs. (*n* = 2,649)	0.94/0.77	0.955 (0.947–0.964)	Ref	—	0.92/0.69	0.921 (0.911–0.932)	Ref	—	0.92/0.74	0.939 (0.929–0.948)	Ref	—
7–12 yrs. (*n* = 3,179)	0.90/0.81	0.934 (0.924–0.944)	0.002	Ref	0.88/0.72	0.897 (0.885–0.909)	0.002	Ref	0.89/0.77	0.919 (0.908–0.930)	0.007	Ref
>12 yrs. (*n* = 1,153)	0.85/0.93	0.922 (0.903–0.942)	0.002	0.287	0.84/0.77	0.883 (0.860–0.906)	0.003	0.288	0.82/0.86	0.903 (0.882–0.924)	0.003	0.192

*MoCA, Montreal Cognitive Assessment; s-MoCA, short forms of the standard MoCA; Se, sensitivity; Sp, specificity; AUC, areas under the receiver operating characteristic curves; CI, confidence interval; Ref, reference group. P-value was based on the DeLong test, a method to compare two AUCs*.

## Discussion

In this study, two short variants of the MoCA were constructed using IRT and CAT in a large sample. The results indicated that the standard MoCA and s-MoCA scores were significantly lower in affected individuals than in unaffected individuals. These s-MoCAs had high diagnostic accuracy across neurological disorders, including different types of dementia and MCI. The overall high sensitivity and specificity may be because the items selected for the s-MoCA span several neurocognitive domains.

Our short variants of the MoCA included the digit span item, which was most likely to be omitted from the published short forms (Liew, 2019; McDicken et al., 2019). Moreover, our abbreviated MoCAs removed the orientation and fluency items (Liew, 2019; McDicken et al., 2019), which were prevalent in the published short variants. These findings indicate that a culturally specific Chinese s-MoCA is needed.

Episodic memory loss is a hallmark clinical symptom associated with AD and aMCI (Albert, 2011). The most discriminative item of the MoCA for identifying memory loss is delayed recall, which is included in our short variants of the MoCA and all current abbreviated MoCAs published (Liew, 2019; McDicken et al., 2019).

Deficits in executive function are the hallmark cognitive dysfunction in patients with VD (O'Brien and Thomas, 2015), frontotemporal dementia (FTD) (Bang et al., 2015), dementia with Lewy bodies (DLB) (Aldridge et al., 2018), Parkinson's disease dementia (PDD) (Aldridge et al., 2018) and naMCI (Chung et al., 2019). The most effective items on the MoCA for screening impairments in executive function include trail making and clock draw, which were items selected for inclusion in the short variants in this study and the recently published s-MoCAs (Roalf et al., 2016; Bezdicek et al., 2018).

Serial subtraction, which is a measure of complex attention, is identified as an item that differentiates between AD and MCI individuals and individuals with MCI from healthy individuals on the MoCA. This item is included in our s-MoCA and many short variants of the MoCA (Horton et al., 2015; Roalf et al., 2016; Bezdicek et al., 2018).

We also observed that the inclusion of digit span and abstraction re-emphasizes the sensitivity of the s-MoCA. This is in accordance with the results of the study by Bezdicek et al. (2018), which indicated that digit span (backwards) and abstraction (watch) were useful in the differentiation of cognitive impairment from HCs.

Both the standard MoCA and short forms of the MoCA in our study highly differentiated the affected individuals from the HCs and had comparable AUCs ≥ 0.88. The optimal cutoff scores of these two short MoCA tests were ≤ 10 and ≤ 12. These tests demonstrated a sensitivity of 0.89/0.90 and specificity of 0.72/0.77, which is similar in performance to the MoCA in identifying cognitive impairment (sensitivity 0.91, specificity 0.82). Both levels were higher than those in the majority of published studies (Bezdicek et al., 2018; McDicken et al., 2019). In those papers where short forms of the MoCA were used to diagnose MCI in older adults (Wittich et al., 2010; Horton et al., 2015; Cecato et al., 2016; Roalf et al., 2016; Larner, 2017; Bezdicek et al., 2018), the median sensitivity across studies was 0.82 (range: 0.44–0.93), and the specificity was 0.69 (0.60–0.98). In addition, the 4- or 6-item versions of the s-MoCA can be administered in ~5 min. Therefore, while the administration of the full MoCA is limited by time constraints in routine clinical practice, the s-MoCA may serve as a viable alternative for the detection of MCI in the elderly population.

In this study, MoCA and s-MoCA showed a lower diagnostic accuracy in younger individuals (60–79 years old) or highly educated participants (≥7 years). Previously published studies indicated that MoCA demonstrated varied diagnostic performance in different age and education groups (Nasreddine et al., 2005; Tan et al., 2015). The hypothesis of brain reserve and cognitive reserve (CR) can be a tentative explanation of these findings. Brain reserve refers to brain volume and the number of connections among neurons, and those with younger age tended to have high brain reserve (Amanollahi et al., 2021). CR is believed to counter the effects of aging or brain damage, and higher education is associated with higher CR (Amanollahi et al., 2021). Individuals with high brain reserve or CR, can compensate cognitive decline (Amanollahi et al., 2021). And younger individuals or highly educated participants were less likely to be identified as cognitively impaired through cognitive tests. Thus, the discriminative ability for the detection of cognitive impairment in these populations was reduced.

Regarding the fact that the risk factors for dementia are an older age and lower education level (Tan et al., 2015), individuals aged over 80 years and less-educated individuals are at an increased risk of developing dementia, and adequate screening for cognitive impairment is vital. Both the full MoCA and s-MoCA are highly reliable in these populations. These results present an important finding because published studies lack population-based data on individuals older than 80 years (Wittich et al., 2010; Horton et al., 2015; Cecato et al., 2016; Roalf et al., 2016; Larner, 2017; Bezdicek et al., 2018) and less-educated individuals (Horton et al., 2015; Roalf et al., 2016). As a result, these short variants of the MoCA may serve as quick and effective instruments for screening for cognitive impairment among individuals with an older age and lower education level.

This study has some strengths. We developed short variants of MoCA and evaluated their utility and sensitivity in the largest sample to date in the literature (*n* = 6,981). Second, we adopted a standardized scientific diagnostic process to obtain a correct diagnosis of cognitive status. In addition, the short variants were developed through more rigorous methods, i.e., IRT and CAT, in a large broad neurological sample. Hence, the selection of items within the original MoCA that are most discriminative of participants with varying cognitive function was allowed.

This study also has limitations. More than 95% of the participants in this study were male, and the recruited population were veterans, which limited the generalizability of the findings to other populations. Directions for future research include the development of abbreviated MoCAs in various clinical settings and populations with more balanced sex ratios. Second, the age structure of the subjects in this study is characterized by the large proportion of the population in the older age groups (≥ 80 years old). This leads to one limitation of the study, which is the lack of people under 60 years of age. However, this study provided data on individuals aged ≥90 years. Third, similar to the development of many abbreviated test versions, the s-MoCA was derived from the administration of the standard version. Hence, the short form was not administered as a unique test, and the total administration time has not yet been determined. In addition, the diagnosis accuracy of s-MoCA requires a further external validation.

## Conclusions

In conclusion, these Chinese versions of the s-MoCA preserved the discriminative validity of the full MoCA and have comparable diagnostic accuracy in the differentiation of individuals with different types of MCI and dementia from HCs similar to the standard MoCA, especially in the old-old and less-educated populations. Therefore, the Chinese s-MoCA may serve as a more rapid and effective instrument in the screening for cognitive impairment when the original MoCA cannot be feasibly administered in clinical services with a high patient load and limited resources for cognitive testing (such as in primary care and geriatric clinics).

## Data Availability Statement

The original contributions presented in the study are included in the article/Supplementary Material, further inquiries can be directed to the corresponding author/s.

## Ethics Statement

The studies involving human participants were reviewed and approved by The Institutional Review Board of the General Hospital of the People's Liberation Army. The patients/participants provided their written informed consent to participate in this study.

## Author Contributions

J-pT: designed and carried out the study, collected the data, and reviewed and revised the manuscript. XW: conducted the statistical analysis and drafted, and reviewed and revised the manuscript. SZ: carried out the study, collected the data, and reviewed and revised the manuscript. YZ: designed the study and coordinated and supervised the data collection. XL: carried out the study and collected the data. NL: conceptualized and designed the study and reviewed and revised the manuscript. L-nW and JG: conceptualized and designed the study, coordinated and supervised the data collection, and critically reviewed and revised the manuscript. All authors contributed to the article and approved the submitted version.

## Conflict of Interest

The authors declare that the research was conducted in the absence of any commercial or financial relationships that could be construed as a potential conflict of interest.

## Supplementary Material

Supplementary Figure 1 Parallel analysis scree plot of the MoCA. The large ratio between the first and second eigenvalues (4.05) suggests that the MoCA is unidimensional enough for IRT. MoCA, Montreal Cognitive Assessment; IRT, item response theory.

Supplementary Figure 2 Residual correlations produced by the confirmatory factor analysis. Residual correlations smaller than 0.2 support the local independence assumption.

Supplementary Figure 3 Item characteristic curves of the MoCA subitems. MoCA, Montreal Cognitive Assessment. The probability of endorsing an item increases as an individual's trait level increases, indicating the assumption of monotonicity.
