# Supplementary figures and images for: Accuracy of the Short-Form Montreal Cognitive Assessment Chinese Versions
Source: Front Aging Neurosci. 2021 Jun 22;13:687824. doi: 10.3389/fnagi.2021.687824 (PMC8258379; doi:10.3389/fnagi.2021.687824)

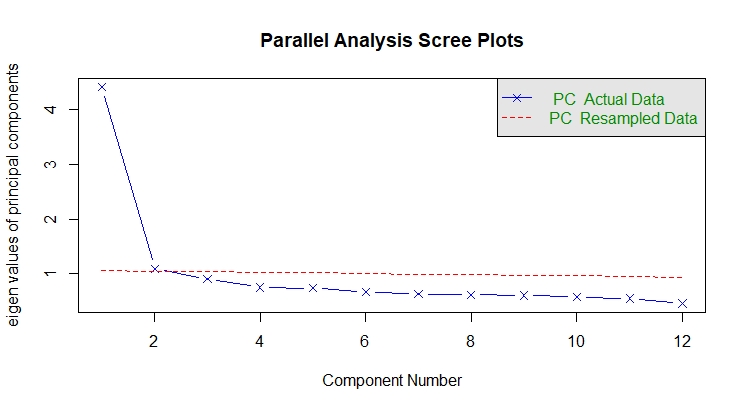

Supplement: Supplementary Figure 1 — Parallel analysis scree plot of the MoCA. The large ratio between the first and second eigenvalues (4.05) suggests that the MoCA is unidimensional enough for IRT. MoCA, Montreal Cognitive Assessment; IRT, item response theory. [file Image_1.JPEG]

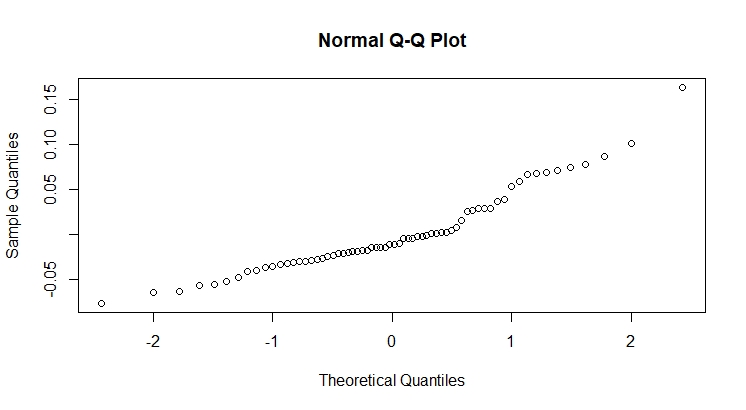

Supplement: Supplementary Figure 2 — Residual correlations produced by the confirmatory factor analysis. Residual correlations smaller than 0.2 support the local independence assumption. [file Image_2.JPEG]

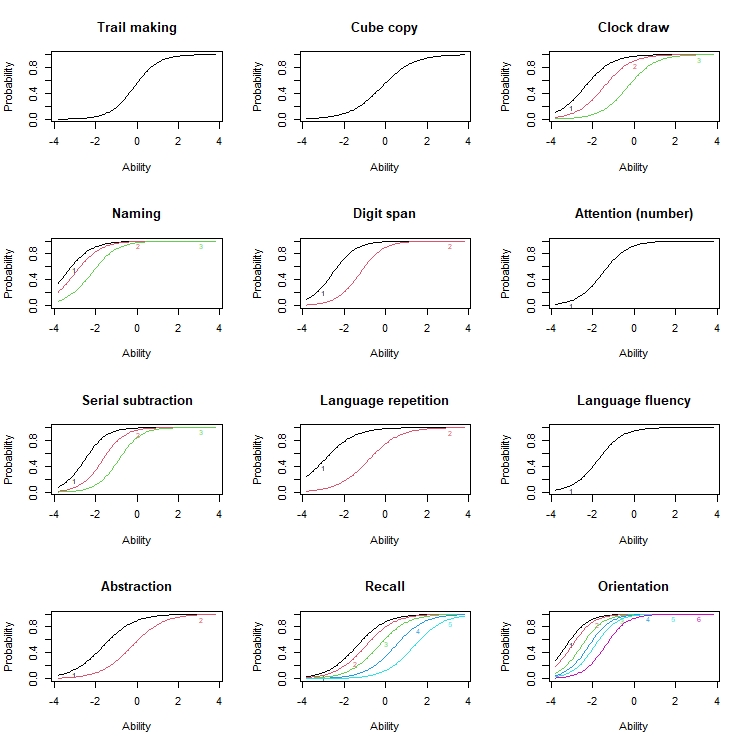

Supplement: Supplementary Figure 3 — Item characteristic curves of the MoCA subitems. MoCA, Montreal Cognitive Assessment. The probability of endorsing an item increases as an individual's trait level increases, indicating the assumption of monotonicity. [file Image_3.JPEG]
